# ECDC’s latest publications

**DOI:** 10.2807/1560-7917.ES.2017.22.26.30555

**Published:** 2017-06-29

**Authors:** Eurosurveillance editorial team

**Affiliations:** 1 European Centre for Disease Prevention and Control (ECDC), Stockholm, Sweden

**Keywords:** infectious diseases, public health, Europe

**Rapid risk and outbreak assessments**

Multi-country outbreak of new Salmonella enterica 11:z41:e,n,z15 infections associated with sesame seeds

Date of publication: 14 June 2017

http://ecdc.europa.eu/en/publications/Publications/rapid-outbreak-assessment-salmonella-enterica-Greece-14-Jun-2017.pdf

Increase of cholera cases in the Horn of Africa and the Gulf of Aden - risk for EU/EEA citizens

Date of publication: 23 May 2017

http://ecdc.europa.eu/en/publications/_layouts/forms/Publication_DispForm.aspx?List=4f55ad51-4aed-4d32-b960-af70113dbb90&ID=1703

Hepatitis A outbreaks in the EU/EEA mostly affecting men who have sex with men

Date of publication: 19 May 2017

http://ecdc.europa.eu/en/publications/_layouts/forms/Publication_DispForm.aspx?List=4f55ad51-4aed-4d32-b960-af70113dbb90&ID=1701

Outbreak of Ebola virus disease in Bas Uele province, Democratic Republic of Congo

Date of publication: 19 May 2017

http://ecdc.europa.eu/en/publications/_layouts/forms/Publication_DispForm.aspx?List=4f55ad51-4aed-4d32-b960-af70113dbb90&ID=1699

Potential public health risks related to communicable diseases at the WorldPride festival in Madrid, 23 June-2 July 2017

Date of publication: 8 May 2017

http://ecdc.europa.eu/en/publications/_layouts/forms/Publication_DispForm.aspx?List=4f55ad51-4aed-4d32-b960-af70113dbb90&ID=1697

Risk related to the use of 'do-it-yourself' CRISPR-associated gene engineering kit comtaminated with pathogenic bacteria

Date of publication: 2 May 2017

http://ecdc.europa.eu/en/publications/_layouts/forms/Publication_DispForm.aspx?List=4f55ad51-4aed-4d32-b960-af70113dbb90&ID=1692

Outbreak of yellow fever in Brazil

Date of publication: 14 April

http://ecdc.europa.eu/en/publications/_layouts/forms/Publication_DispForm.aspx?List=4f55ad51-4aed-4d32-b960-af70113dbb90&ID=1675

Multidrug-resistant tuberculosis in migrants (MDR TB), multi-country cluster

Date of publication: 13 April 2017

http://ecdc.europa.eu/en/publications/_layouts/forms/Publication_DispForm.aspx?List=4f55ad51-4aed-4d32-b960-af70113dbb90&ID=1673

Zika virus disease epidemic

Date of publication: 4 April

http://ecdc.europa.eu/en/publications/_layouts/forms/Publication_DispForm.aspx?List=4f55ad51-4aed-4d32-b960-af70113dbb90&ID=1671

**Technical reports**

Vaccine-preventable diseases and immunisation: Core competencies

Date of publication: 14 June 2017

http://ecdc.europa.eu/en/publications/Publications/VPD%20Competencies%20Training_Short_Technical%20report_final.pdf

Euro-GASP external quality assessment (EQA) scheme for Neisseria gonorrhoeae antimicrobial susceptibility testing 2016

Date of publication: 7 June 2017

http://ecdc.europa.eu/en/publications/Publications/EQA%20Report%202016%20final.pdf

A literature review of Training Needs Assessment (TRNA) methodology

Date of publication: 1 June 2017

http://ecdc.europa.eu/en/publications/Publications/LR-TRNA_Methodology_25042017_Final.pdf

Hepatitis B and C testing activities, needs, and priorities in the EU/EEA

Date of publication: 5 May 2017

http://ecdc.europa.eu/en/publications/_layouts/forms/Publication_DispForm.aspx?List=4f55ad51-4aed-4d32-b960-af70113dbb90&ID=1706

EU Laboratory Capability Monitoring System (EULabCap): report on 2015 survey of EU/EEA country capabilities and capacities

Date of publication: 28 April 2017

http://ecdc.europa.eu/en/publications/_layouts/forms/Publication_DispForm.aspx?List=4f55ad51-4aed-4d32-b960-af70113dbb90&ID=1690

Immunisation information systems in the EU and EEA - results of a survey on implementation and system characteristics

Date of publication: 26 April 2017

http://ecdc.europa.eu/en/publications/Publications/immunisation-systems.pdf

Catalogue of interventions addressing vaccine hesitancy

Date of publication: 25 April 2017

http://ecdc.europa.eu/en/publications/_layouts/forms/Publication_DispForm.aspx?List=4f55ad51-4aed-4d32-b960-af70113dbb90&ID=1683

Economic evaluations of interventions to prevent healthcare-associated infections - literature review

Date of publication: 19 April 2017

http://ecdc.europa.eu/en/publications/_layouts/forms/Publication_DispForm.aspx?List=4f55ad51-4aed-4d32-b960-af70113dbb90&ID=1676

**Scientific Advice**

Systematic review on the diagnosis, treatment, care and prevention of tuberculosis in prison settings

Date of publication: 30 May 2017

http://ecdc.europa.eu/en/publications/_layouts/forms/Publication_DispForm.aspx?List=4f55ad51-4aed-4d32-b960-af70113dbb90&ID=1707

**Special report**

Monitoring implementation of the Dublin Declaration on partnership to fight HIV/AIDS in Europe and Central Asia: 2017 progress report

Date of publication: 26 April 2017

HIV and men who have sex with men: http://ecdc.europa.eu/en/publications/_layouts/forms/Publication_DispForm.aspx?List=4f55ad51-4aed-4d32-b960-af70113dbb90&ID=1688
Continuum of HIV care: http://ecdc.europa.eu/en/publications/_layouts/forms/Publication_DispForm.aspx?List=4f55ad51-4aed-4d32-b960-af70113dbb90&ID=1682
HIV testing: http://ecdc.europa.eu/en/publications/_layouts/forms/Publication_DispForm.aspx?List=4f55ad51-4aed-4d32-b960-af70113dbb90&ID=1685
HIV and migrants: http://ecdc.europa.eu/en/publications/_layouts/forms/Publication_DispForm.aspx?List=4f55ad51-4aed-4d32-b960-af70113dbb90&ID=1686
HIV and treatment:
http://ecdc.europa.eu/en/publications/_layouts/forms/Publication_DispForm.aspx?List=4f55ad51-4aed-4d32-b960-af70113dbb90&ID=1684

**Technical document**

Surveillance of healthcare-associated infections and prevention indicators in European intensive care units - HAI-Net ICU protocol version 2.2  Date of publication: 5 May 2017

http://ecdc.europa.eu/en/publications/_layouts/forms/Publication_DispForm.aspx?List=4f55ad51-4aed-4d32-b960-af70113dbb90&ID=1694

Surveillance of surgical site infections and prevention indicators in European hospitals - HAI-Net SSI protocol version 2.2

Date of publication: 5 May 2017

http://ecdc.europa.eu/en/publications/_layouts/forms/Publication_DispForm.aspx?List=4f55ad51-4aed-4d32-b960-af70113dbb90&ID=1695

European surveillance of *Clostridium difficile* infections - surveillance protocol version 2.3

Date of publication: 21 April 2017

http://ecdc.europa.eu/en/publications/_layouts/forms/Publication_DispForm.aspx?List=4f55ad51-4aed-4d32-b960-af70113dbb90&ID=1677

**Mission report**

Technical mission: HIV, STI and viral hepatitis in Bulgaria. 19-21 September 2016 and 14-15 November 2016

Date of publication: 29 May 2017

http://ecdc.europa.eu/en/publications/_layouts/forms/Publication_DispForm.aspx?List=4f55ad51-4aed-4d32-b960-af70113dbb90&ID=1705

**Surveillance reports**

Legionnaires’ disease in Europe, 2015

Date of publication: 14 June 2017

http://ecdc.europa.eu/en/publications/Publications/Legionnares-disease-europe-2015.pdf

Measles and rubella monitoring, January 2017

Date of publication: 24 April 2017

http://ecdc.europa.eu/en/publications/_layouts/forms/Publication_DispForm.aspx?List=4f55ad51-4aed-4d32-b960-af70113dbb90&ID=1679

